# An enriched environment ameliorates maternal sleep deprivation-induced cognitive impairment in aged mice by improving mitochondrial function via the Sirt1/PGC-1α pathway

**DOI:** 10.18632/aging.205385

**Published:** 2024-01-16

**Authors:** Ru-Meng Wei, Yue-Ming Zhang, Kai-Xuan Zhang, Gao-Xia Liu, Xue-Yan Li, Jing-Ya Zhang, Wei-Zhong Lun, Xue-Chun Liu, Gui-Hai Chen

**Affiliations:** 1 Department of Neurology (Sleep Disorders), The Affiliated Chaohu Hospital of Anhui Medical University, Hefei 238001, Anhui, China; 2 Department of Neurology, The Second People’s Hospital of Hefei, Hefei Hospital Affiliated to Anhui Medical University, Hefei 230011, Anhui, China

**Keywords:** maternal sleep deprivation, environmental enrichment, Sirt1/PGC-1α, neuroinflammation, synaptic dysfunction

## Abstract

Background: Early life stress can cause cognitive impairment in aged offspring. Environmental enrichment (EE) is considered to be an effective non-pharmacological treatment for improving cognitive decline. The aim of this research was to evaluate the effect of EE, on cognitive impairment in aged offspring induced by maternal sleep deprivation (MSD) and the underlying mechanisms involved to investigate its potential value in clinical practice.

Methods: CD-1 damns were subjected or not to sleep deprivation during late gestation. Twenty-one days after birth, the offspring were assigned to standard or EE cages. At 18 months-old, the learning and memory function of the offspring mice was evaluated using Morris water maze. The hippocampal and prefrontal cortical levels of protein, gene, proinflammation cytokines, and oxidative stress indicators was examined by Western blot, real-time polymerase chain reaction, enzyme linked immunosorbent assay, and biochemical assays.

Results: Offspring in MSD group exhibited declined learning and memory abilities compared with control animals. Moreover, the hippocampal and prefrontal cortical levels of Sirtuin1 (Sirt1), peroxisome proliferator-activated receptor-gamma coactivator-1 alpha (PGC-1α), postsynaptic density protein-95, and synaptophysin were lower and those of proinflammation cytokines higher in the MSD group; meanwhile, the superoxide dismutase content was higher and the malondialdehyde and reactive oxygen species contents were lower. However, these deleterious changes were ameliorated by exposure to EE.

Conclusions: EE attenuates MSD-induced cognitive impairment, oxidative stress, and neuroinflammation and reverses the reduction in synaptic protein levels in aged offspring mice via the Sirt1/PGC-1α pathway.

## INTRODUCTION

Sleep is a basic condition essential for human health [1]. However, owing to changes in anatomy and hormone levels, as well as because of psychological factors, pregnant women are more susceptible to sleep dysfunction [2], especially during late pregnancy [3]. Sleep inadequate during late pregnancy not only increases the risk of hypertension and postpartum depression in pregnant women but also has a negative effect on the development of offspring [4]. Several basic studies have documented that sleep deprivation (SD) during pregnancy in rodents can affect the neurodevelopment of the offspring, manifesting as cognitive impairment, increase in anxiety-like behaviors, and immature sleep patterns [5, 6].

Maternal sleep deprivation (MSD) can lead to the reprogramming of the immune cells of the brain in utero, disrupt neurogenesis, impair synaptic plasticity, and activate microglia in later life, subsequently increasing the risk of cognitive impairment in the offspring during adolescence or adulthood [6–8]. However, relatively few studies have explored the effects of MSD on cognitive function in aging offspring, including the underlying mechanisms. We previously provided preliminary evidence indicating that the adverse effects of late-pregnancy SD on cognitive function in offspring can persist into old age, with mechanisms involving neuroinflammation [9]. However, the mechanisms responsible for cognition impairment in aged offspring induced by SD during late pregnancy remain largely unknown.

The maintenance and regulation of cognitive function largely rely on two critical brain regions, namely, the prefrontal cortex and the hippocampus [10]. The brain is susceptible to oxidative stress owing to its high oxygen consumption, high polyunsaturated fatty acid content, and low antioxidant defense capacity [11]. Growing evidence suggests that cognitive dysfunction is closely associated with oxidative stress in the brain. Indeed, neurodegenerative diseases including Alzheimer’s disease (AD), Parkinson’s disease, and amyotrophic lateral sclerosis are accompanied by a significant increase in oxidative stress, as showed by the elevated expression levels of reactive oxygen species (ROS) and malondialdehyde (MDA) and the decrease in the contents of antioxidant enzymes such as superoxide dismutase (SOD) and glutathione peroxidase [12, 13]. Inflammation also plays an important role in cognitive disorders. Preclinical and clinical studies have both shown that cognitive impairment induced by AD is accompanied by elevated levels of pro-inflammatory cytokines including interleukin-1 beta (IL-1β), IL-6, and tumor necrosis factor-alpha (TNF-α) [14, 15]. Together, these evidences suggest that oxidative stress and inflammation may be important mechanisms leading to cognitive impairment.

Mitochondrial biogenesis disruption is closely related to oxidative stress and neuroinflammation [16]. Peroxisome proliferator-activated receptor-gamma coactivator-1 alpha (PGC-1α) is a transcription factor involved in the regulation of mitochondrial biogenesis and antioxidant damage in the nervous system, thus contributing to the regulation of cognitive impairment induced by various neurodegenerative diseases [17]. The transcription factor Sirtuin1 (Sirt1) can activate PGC-1α by deacetylation, resulting in increased synthesis and secretion of synaptic proteins in the brain, and thereby mitigating cognitive decline [18, 19]. The activated Sirt1/PGC-1α pathway can improve cognitive deficits induced by chronic cerebral hypoperfusion, scopolamine, or a high-fat diet by modulating oxidative stress and neuroinflammation [18–20]. This suggests that the Sirt1/PGC-1α pathway has potential as a therapeutic target for improving cognitive impairment induced by SD in late pregnancy. Treatment for cognitive impairment in older offspring is critical to the health of older patients. However, no drugs with a clear therapeutic target have been identified, and therefore, non-pharmacological treatments have the potential to be a promising therapeutic modality.

Environmental enrichment (EE) is an effective and simple non-pharmacological intervention for improving cognitive dysfunction by providing increased opportunities for social interaction, exercise, and somatosensory stimulation [21]. EE can not only alleviate or even prevent cognitive dysfunction in normal aging [22] but can also counteract cognitive deficits caused by early-life stress [23]. The mechanisms by which EE ameliorates cognitive deficits, at least in part, were through improvements in neuroinflammation, oxidative stress, and mitochondrial function, as well as increases in hippocampal synaptic plasticity [24–26]. Our preliminary results suggested that cognitive impairment in aging offspring resulting from MSD can be alleviated by long-term EE [9]. However, how EE affects cognitive impairment in aging offspring resulting from gestational exposure to SD is unclear, as are the putative accompanying changes in mitochondrial function, oxidative stress, and the Sirt1/PGC-1α pathway.

In this study, we explored whether SD during late pregnancy accelerates cognitive decline in aging offspring mice and, if so, whether long-term EE can ameliorate cognitive impairment through improving oxidative stress and neuroinflammation in a Sirt1/PGC-1α pathway-dependent manner.

## RESULTS

### EE improved MSD-induced learning and memory decline

**Figure 1:**
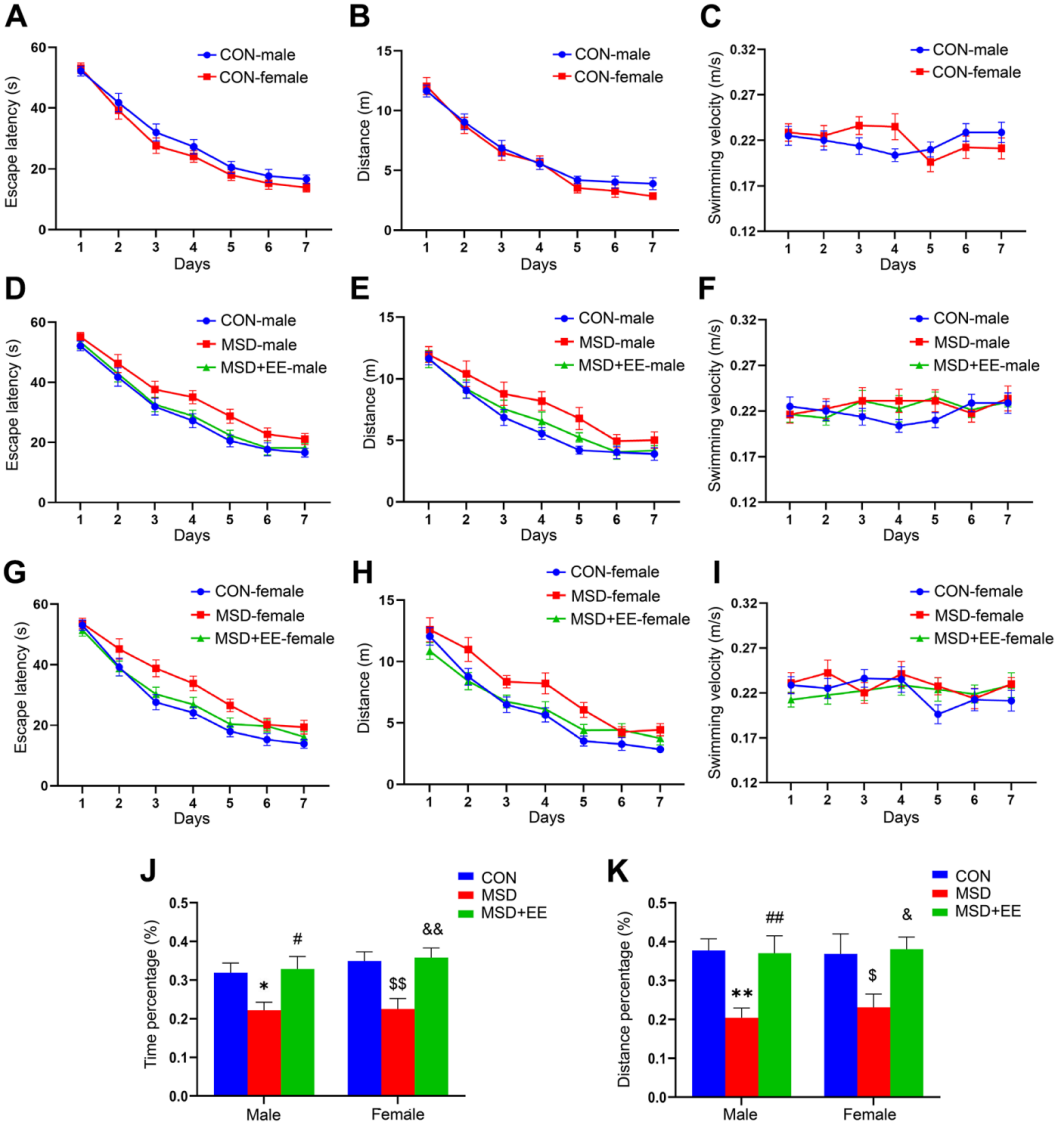
**The effects of EE on MSD-induced changes in cognitive performance (n = 8).** Escape latency, swim distance and velocity of control (CON) group (**A**–**C**), males (**D**–**F**), and females (**G**–**I**). (**J**) Percent time spent in the target quadrant. (**K**) Percent distance swam in the target quadrant. ^*^P < 0.05 and ^**^ *P*< 0.01 vs. male mice in the CON group; ^#^*P* < 0.05 and ^##^*P* < 0.01 vs. male mice in the MSD group; ^$^*P* < 0.05 and ^$$^*P* < 0.01 vs. female mice in the CON group; ^&^*P* < 0.05 and ^&&^*P* < 0.01 vs. female mice in the MSD group.

In the acquisition phase of the MWM test, the results showed no sex differences in escape latency and swim distance for the control and other treatment groups (Figure 1A, 1B and Supplementary Figures 1A, 1B, 2A, 2B). However, marked differences in latency and swim distance were observed between treatments in all three groups (latency: male: *F*_(2, 21)_ = 11.46, *P* < 0.01; female: *F*_(2, 21)_ = 13.68, *P* < 0.01; distance: male: *F*_(2, 21)_ = 10.18; female: *F*_(2, 21)_ = 18.94, *P* < 0.01; Figure 1D, 1E, 1G, 1H). *Post hoc* comparisons showed that the escape latency and swim distance were increased in the MSD group when compared with the Control group (*Ps* < 0.05). However, the escape latency and swim distance were shorter in the MSD+EE group than in the MSD group (*Ps* < 0.05). No difference in swimming velocity among the groups whether controlling for sex or treatment (Figure 1C, 1F, 1I and Supplementary Figures 1C, 2C).

In the memory phase of the MWM test, the percentage of time spent and the distance swam in the target quadrant differed in the three groups (precent of time: *F*_(2, 42)_ = 13.23, *P* < 0.01; percent of distance: *F*_(2, 42)_ = 11.80, *P* < 0.01; Figure 1J, 1K). Furthermore, the time and distance percentages were clearly lower in the MSD group than in controls (*Ps* < 0.05) but markedly higher in the MSD+EE group than in the MSD group (*Ps* < 0.05).

### EE improved MSD-induced oxidative stress

**Figure 2:**
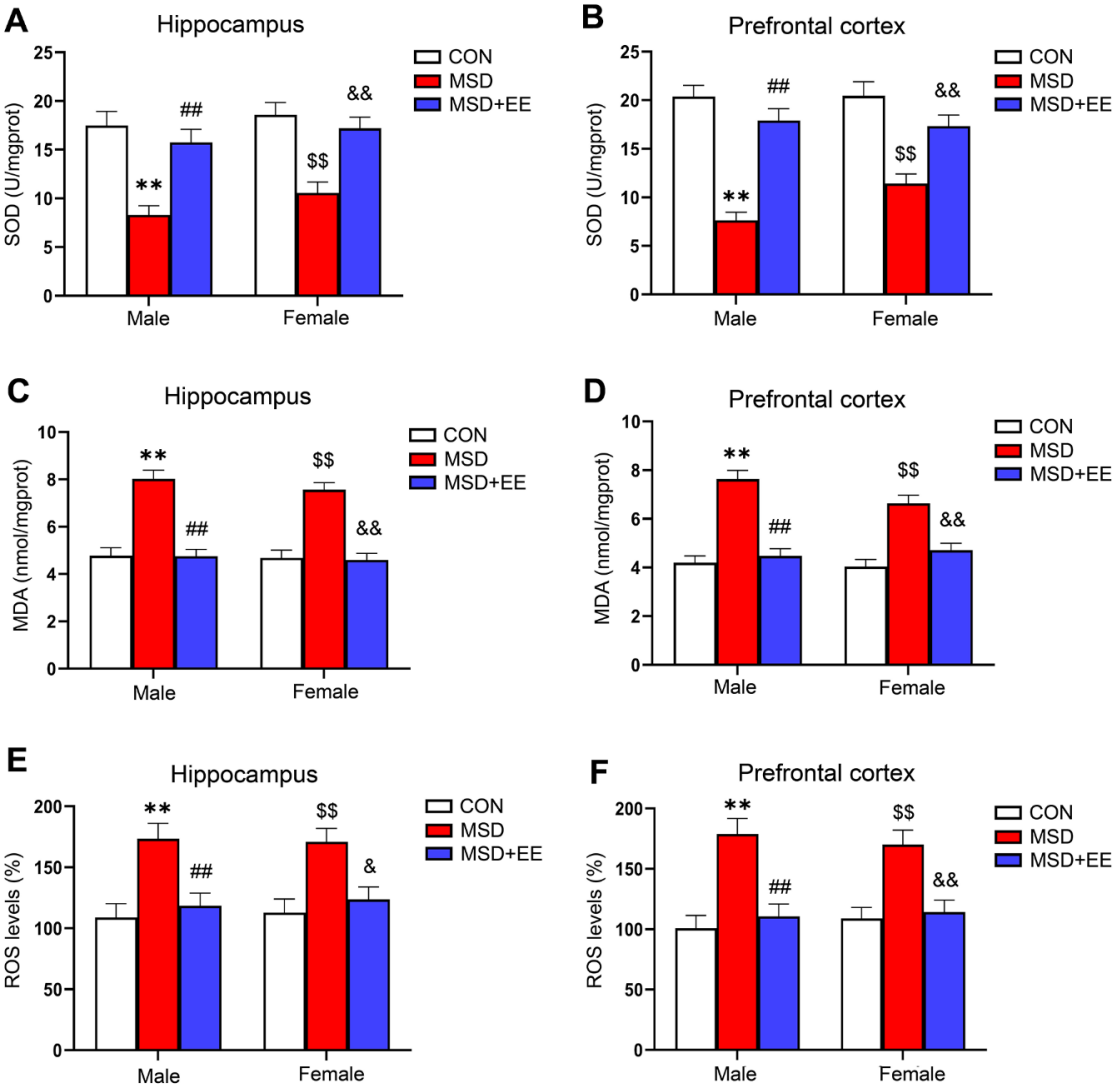
**The effect of EE on change in MSD-induced oxidative stress levels (n = 8).** The level of SOD in the hippocampus (**A**) and prefrontal cortex (**B**). The level of MDA in the hippocampus (**C**) and prefrontal cortex (**D**). The level of ROS in the hippocampus (**E**) and prefrontal cortex (**F**). ^**^P < 0.01 vs. males in the control (CON) group; ^##^*P* < 0.01 vs. males in the MSD group; ^$$^*P* < 0.01 vs. females in the CON group; ^&^*P* < 0.05 and ^&&^*P* < 0.01 vs. females in the MSD group.

In the hippocampus and prefrontal cortex, the levels of SOD, MDA, and ROS were obviously different in all groups (hippocampus: SOD: *F*_(2, 42)_ = 28.73, *P* < 0.01; MDA: *F*_(2, 42)_ = 63.83, *P* < 0.01; ROS: *F*_(2, 30)_ = 17.16, *P* < 0.01; prefrontal cortex: SOD: *F*_(2, 42)_ = 48.86, *P* < 0.01; MDA: *F*_(2, 42)_ = 55.12, *P* < 0.01; ROS: *F*_(2, 30)_ = 24.45, *P* < 0.01; Figure 2A–2F). Compared with the control group, MDA and ROS levels in both brain regions were significantly increased in the MSD group, whereas SOD levels were decreased (*Ps* < 0.05). However, MDA and ROS levels were lower in the MSD+EE group than in the MSD group in both brain regions, whereas the opposite was observed for SOD content (*Ps* < 0.05).

### EE alleviated MSD-induced inflammation

**Figure 3:**
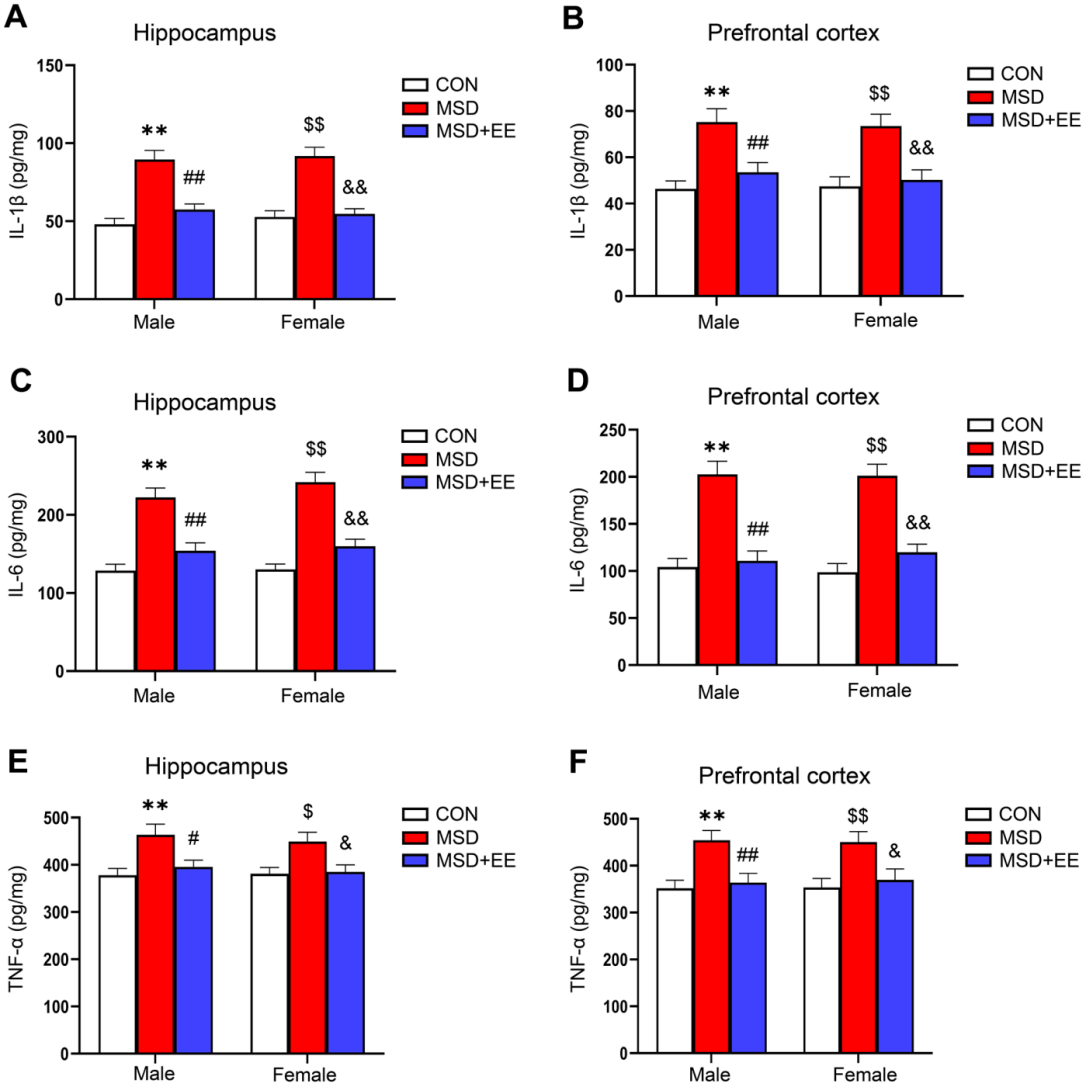
**The effect of EE on the increased levels of proinflammation cytokines of aging mice induced by MSD (n = 8).** The level of IL-1β in the hippocampus (**A**) and prefrontal cortex (**B**). The level of IL-6 in the hippocampus (**C**) and prefrontal cortex (**D**). The level of TNF-α in the hippocampus (**E**) and prefrontal cortex (**F**). ^**^P < 0.01 vs. males in the control (CON) group; ^#^*P* < 0.05 and ^##^*P* < 0.01 vs. males in the MSD group; ^$^*P* < 0.05 and ^$$^*P* < 0.01 vs. females in the CON group; ^&^*P* < 0.05 and ^&&^*P* < 0.01 vs. females in the MSD group.

In the hippocampus and prefrontal cortex, the IL-1β, IL-6, and TNF-α levels differed among the groups (hippocampus: IL-1β: *F*_(2, 42)_ = 48.44, *P* < 0.01; IL-6: *F*_(2, 42)_ = 57.38, *P* < 0.01; TNF-α: *F*_(2, 42)_ = 12.52, *P* < 0.01; prefrontal cortex: IL-1β: *F*_(2, 42)_ = 20.42, *P* < 0.01; IL-6: *F*_(2, 42)_ = 51.13, *P* < 0.01; TNF-α: *F*_(2, 42)_ = 13.67, *P* < 0.01; Figure 3A–3F). Additionally, the IL-1β, IL-6, and TNF-α levels were increased in the MSD group relative to Control group (*Ps* < 0.05). Nevertheless, EE significantly mitigated these increases (*Ps* < 0.05).

### EE decreased the mRNA level of Iba-1 and increased those of SYN, PSD-95, PGC-1α, and Sirt1 in the hippocampus

**Figure 4:**
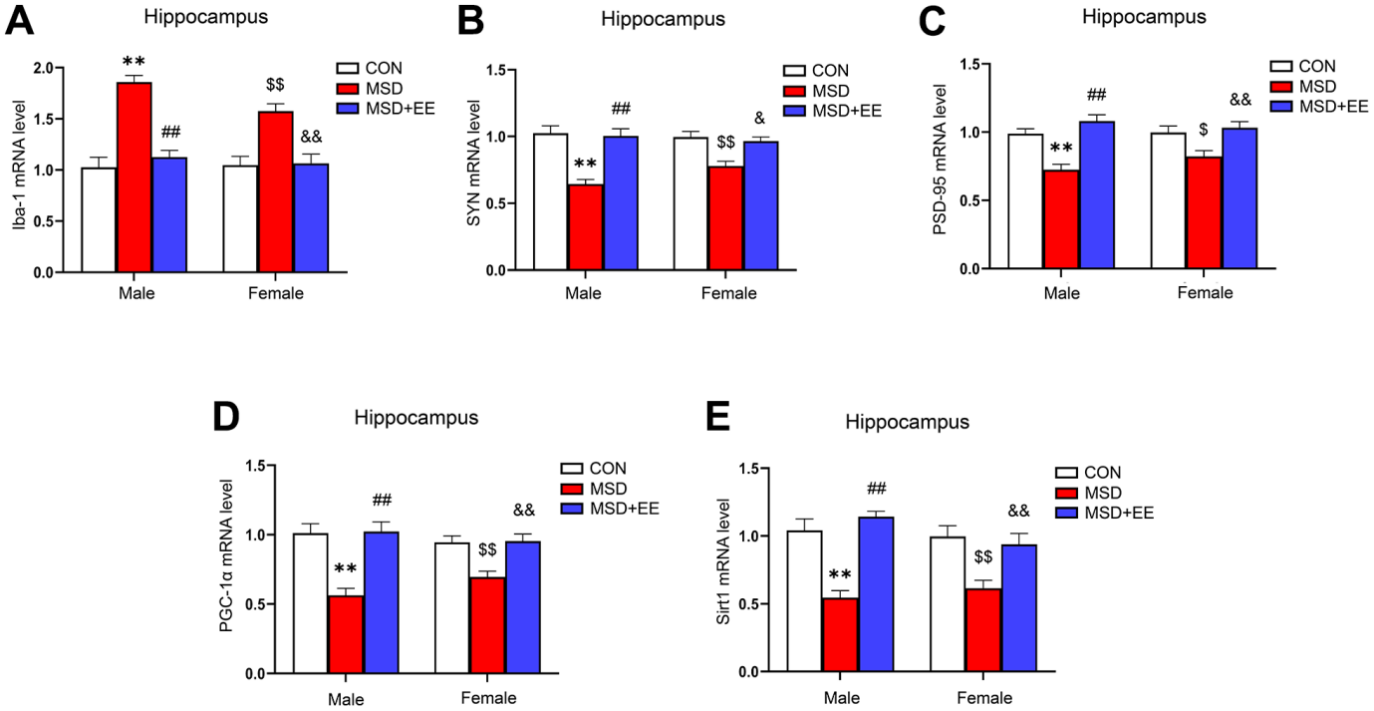
**The effects of EE on MSD-induced alterations in Iba-1, SYN, PSD-95, PGC-1α, and Sirt1 levels in the hippocampus of aging mice (n = 8).** The Iba-1 (**A**), SYN (**B**), PSD-95 (**C**), PGC-1α (**D**), and Sirt1 (**E**) mRNA levels in the hippocampus. ^**^P < 0.01 vs. males in the control (CON) group; ^##^*P* < 0.01 vs. males in the MSD group; ^$^*P* < 0.05 and ^$$^*P* < 0.01 vs. females in the CON group; ^&^*P* < 0.05 and ^&&^*P* < 0.01 vs. females in the MSD group.

There were remarkable differences in hippocampal ionized calcium-binding adapter molecule 1 (Iba-1), synaptophysin (SYN), postsynaptic density protein-95 (PSD-95), PGC-1α, and Sirt1 mRNA levels between the three groups (Iba-1: *F*_(2, 42)_ = 44.09, *P* < 0.01; SYN: *F*_(2, 42)_ = 29.66, *P* < 0.01; PSD-95: *F*_(2, 42)_ = 23.27, *P* < 0.01; PGC-1α: *F*_(2, 42)_ = 26.91, *P* < 0.01; Sirt1: *F*_(2, 42)_ = 29.17, *P* < 0.01; Figure 4A–4E). Compared with the Control group, the hippocampal mRNA levels of SYN, PSD-95, PGC-1α, and Sirt1 were significantly decreased, whereas that of Iba-1 was increased (*Ps* < 0.05). Furthermore, the mRNA levels of SYN, PSD-95, PGC-1α, and Sirt1 were higher and that of Iba-1 was lower in the MSD+EE group compared to the MSD group (*Ps* < 0.05).

### EE decreased the mRNA level of Iba-1 and increased those of SYN, PSD-95, PGC-1α, and Sirt1 in the prefrontal cortex

**Figure 5:**
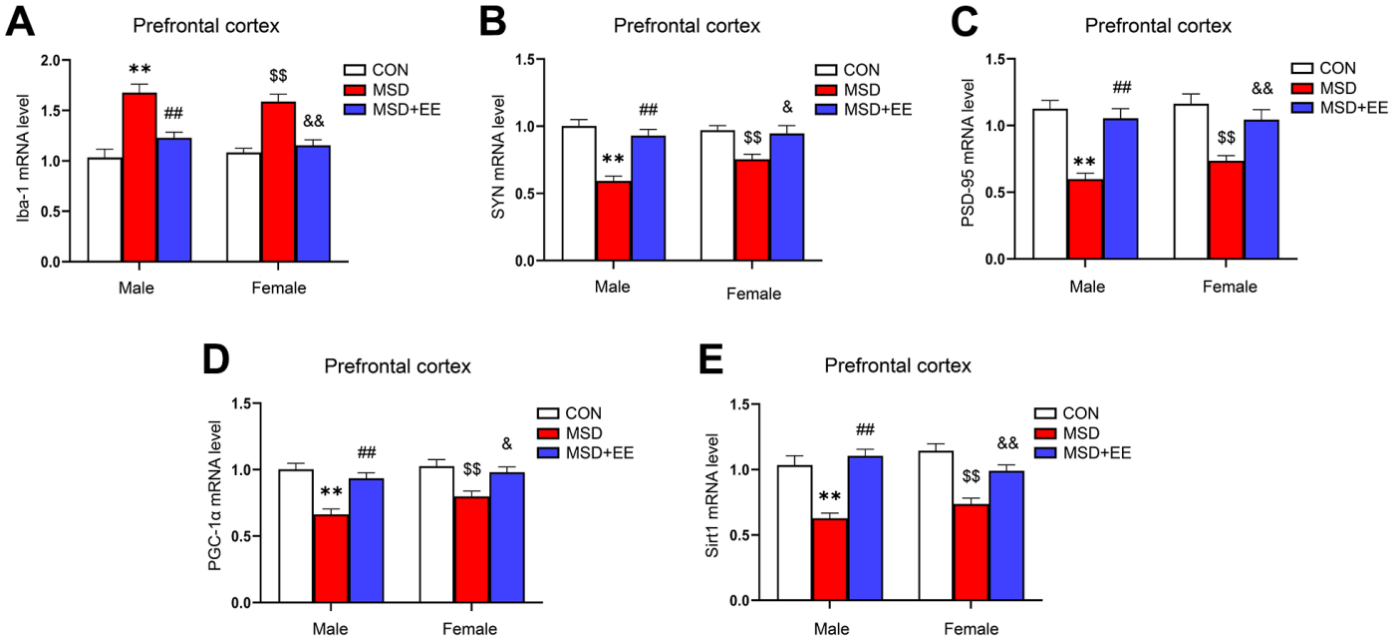
**The effects of EE on MSD-induced alterations in Iba-1, SYN, PSD-95, PGC-1α, and Sirt1 levels in the prefrontal cortex of aging mice (n = 8).** The mRNA levels of Iba-1 (**A**), SYN (**B**), PSD-95 (**C**), PGC-1α (**D**), and Sirt1 (**E**) in the prefrontal cortex. ^**^P < 0.01 vs. males in the control (CON) group; ^##^*P* < 0.01 vs. males in the MSD group; ^$$^*P* < 0.01 vs. females in the CON group; ^&^*P* < 0.05 and ^&&^*P* < 0.01 vs. females in the MSD group.

The mRNA levels of Iba-1, SYN, PSD-95, PGC-1α, and Sirt1 in the prefrontal cortex differed significantly in all groups (Iba-1: *F*_(2, 42)_ = 39.98, *P* < 0.01; SYN: *F*_(2, 42)_ = 28.68, *P* < 0.01; PSD-95: *F*_(2, 42)_ = 31.89, *P* < 0.01; PGC-1α: *F*_(2, 42)_ = 24.17; *P* < 0.01; Sirt1: *F*_(2, 42)_ = 37.12, *P* < 0.01; Figure 5A–5E). The Tukey test indicated that, compared with the controls, the mRNA levels of SYN, PSD-95, PGC-1α, and Sirt1 in the prefrontal cortex were significantly decreased, whereas that of Iba-1 was increased in mice of the MSD group (*Ps* < 0.05). Furthermore, the prefrontal cortical levels of SYN, PSD-95, PGC-1α, and Sirt1 were higher, and that of Iba-1 was lower in the MSD+EE group relative to MSD group (*Ps* < 0.05).

### EE decreased the protein level of Iba-1 and increased those of SYN, PSD-95, PGC-1α, and Sirt1 in the hippocampus

**Figure 6:**
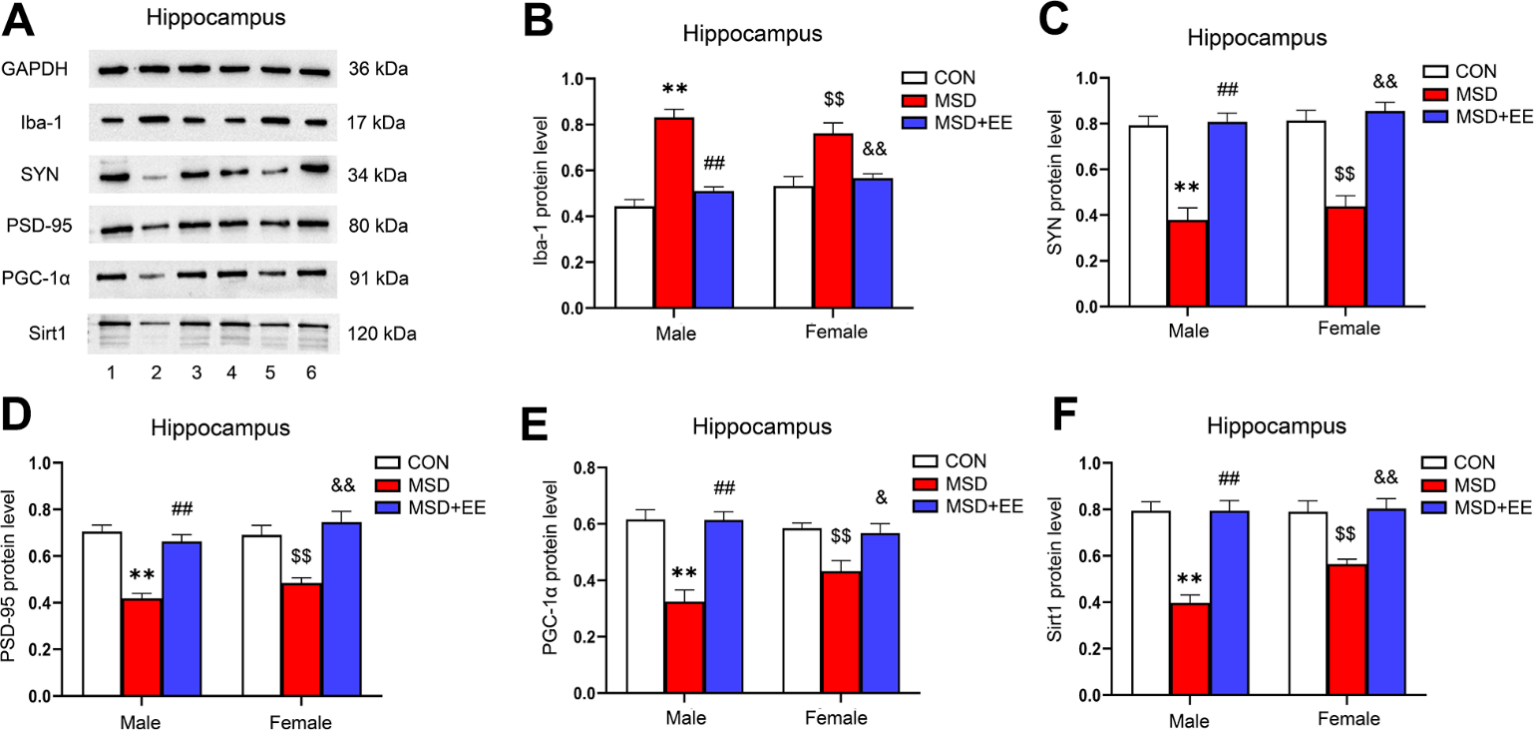
**The effect of EE on MSD-induced changes in hippocampal Iba-1, SYN, PSD-95, PGC-1α, and Sirt1 protein levels (n = 6).** (**A**) The hippocampal expression levels of Iba-1, SYN, PSD-95, PGC-1α, and Sirt1 measured by western blotting. 1: males in the control (CON) group; 2: males in the MSD group; 3: males in the MSD+EE group; 4: females in the CON group; 5: females in the MSD group; 6: females in the MSD+EE group. (**B**–**F**) Protein quantification results. (**B**) Iba-1, (**C**) SYN, (**D**) PSD-95, (**E**) PGC-1α, and (**F**) Sirt1. ^**^P < 0.01 vs. males in the CON group; ^##^*P* < 0.01 vs. males in the MSD group; ^$$^*P* < 0.01 vs. females in the CON group; ^&^*P* < 0.05 and ^&&^*P* < 0.01 vs. females in the MSD group.

There were differences in protein levels of Iba-1, SYN, PSD-95, PGC-1α and Sirt1 in the hippocampus of the three groups (Iba-1: *F*_(2, 30)_ = 48.42, *P* < 0.01; SYN: *F*_(2, 30)_ = 59.18, *P* < 0.01; PSD-95: *F*_(2, 30)_ = 39.13, *P* < 0.01; PGC-1α: *F*_(2, 30)_ = 28.45, *P* < 0.01; Sirt1: *F*_(2, 30)_ = 43.24, *P* < 0.01; Figure 6A–6F). Compared with control animals, the hippocampal protein levels of SYN, PSD-95, PGC-1α, and Sirt1 were significantly decreased in aging MSD-treated mice, whereas that of Iba-1 was increased (*Ps* < 0.05). However, these MSD-induced effects were reversed when mice were exposed to EE (*Ps* < 0.05).

### EE decreased the protein level of Iba-1 and increased those of SYN, PSD-95, PGC-1α, and Sirt1 in the prefrontal cortex

**Figure 7:**
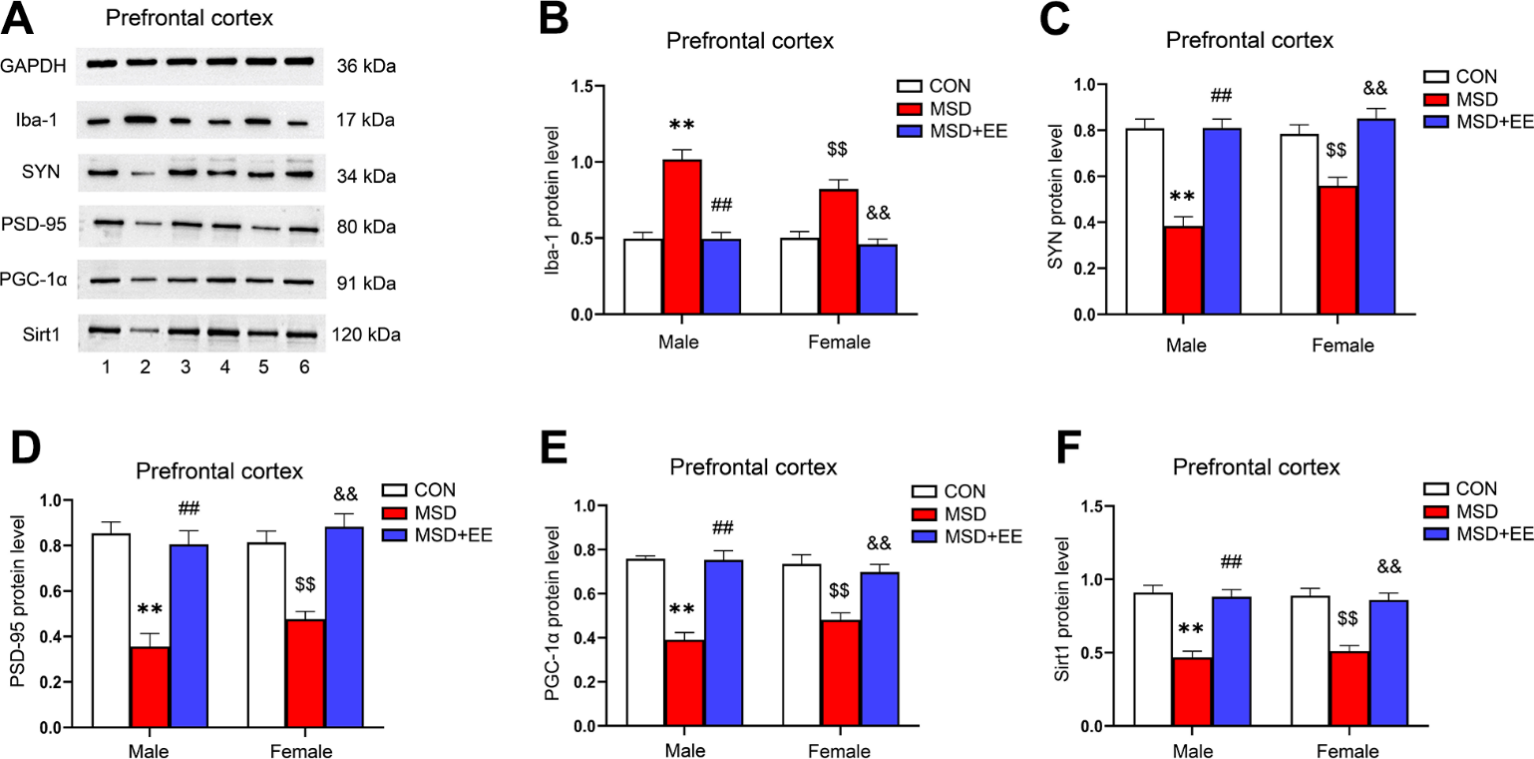
**The effect of EE on MSD-induced changes in prefrontal cortical Iba-1, SYN, PSD-95, PGC-1α, and Sirt1 protein levels (n = 6).** (**A**) The expression levels of Iba-1, SYN, PSD-95, PGC-1α, and Sirt1 in the prefrontal cortex measured by western blotting. 1: males in the control (CON) group; 2: males in the MSD group; 3: males in the MSD+EE group; 4: females in the CON group; 5: females in the MSD group; 6: females in the MSD+EE group. (**B**–**F**) Protein quantification results. (**B**) Iba-1, (**C**) SYN, (**D**) PSD-95, (**E**) PGC-1α, and (**F**) Sirt1. ^**^P < 0.01 vs. males in the CON group; ^##^*P* < 0.01 vs. males in the MSD group; ^$$^*P* < 0.01 vs. females in the CON group; ^&&^*P* < 0.01 vs. females in the MSD group.

The expression levels of Iba-1, SYN, PSD-95, PGC-1α, and Sirt1 in the prefrontal cortex were different in all groups (Iba-1: *F*_(2, 30)_ = 52.42, *P* < 0.01; SYN: *F*_(2, 30)_ = 50.46, *P* < 0.01; PSD-95: *F*_(2, 30)_ = 44.23, *P* < 0.01; PGC-1α: *F*_(2, 30)_ = 52.62, *P* < 0.01; Sirt1: *F*_(2, 30)_ = 48.86, *P* < 0.01; Figure 7A–7F). Comparison results show that the protein levels of SYN, PSD-95, PGC-1α, and Sirt1 in the prefrontal cortex were significantly decreased in the MSD group compared with the Control group, whereas that of Iba-1 was increased (*Ps* < 0.05). However, long-term EE reversed these MSD-mediated changes in the levels of the above proteins (*Ps* < 0.05).

## DISCUSSION

An increasing number of studies have shown that MSD exerts adverse effects on the nervous system of offspring rodents, including immature sleep patterns, an increase in anxiety-like behaviors, and a decrease in cognitive abilities [5, 27, 28]. In this study, we showed that MSD significantly impaired the spatial learning and memory abilities of aged offspring CD-1 mice, increased the levels of Iba-1, pro-inflammatory factors (IL-1β, IL-6, TNF-α), ROS, and MDA, decreased the expression levels of SOD and synaptic proteins (PSD-95, SYN), and inhibited the Sirt1/PGC-1α pathway in the hippocampus and prefrontal cortex. Importantly, EE reversed these adverse effects caused by MSD during late pregnancy by upregulating the Sirt1/PGC-1α pathway, thereby promoting mitochondrial biogenesis.

### EE reversed MSD-induced cognitive impairment

The environment experienced early in life can have lifelong effects on physical health, referred to as the “fetal origins of adult diseases” hypothesis. Specifically, fetal or childhood exposure to adverse stimuli can lead to changes in immune function and epigenetics, as well as the reprogramming of the hypothalamic–pituitary–adrenal axis. These changes can trigger structural or functional alterations in relevant brain regions, leading to depression and cognitive impairment later in life [29, 30]. We have previously revealed that maternal immune stress in late pregnancy exacerbates age-related cognitive impairment in offspring mice [31]. Here, we demonstrated that MSD during late pregnancy leads to cognitive impairment in older offspring mice. EE can improve cognitive function by increasing social, sensory, and spatial complexity [32]. The results of the MWM test exhibited that EE reversed the MSD-induced deficits in cognitive performance in aged offspring, as evidenced by the shorter swim distance and escape latency in the training phase of the test and the increase in the percent distance and time spent in the target quadrant in the memory phase of the MWM test by mice in the MSD+EE group. These findings are in accordance with our previous studies showing that EE improved the cognitive performance in aging mice of spatial learning and memory impairment induced by MSD [9].

### EE relieved oxidative stress and neuroinflammation caused by MSD

Oxidative stress and inflammation are strongly related to cognitive dysfunction [33]. Numerous neurodegenerative diseases are accompanied by elevated oxidative stress [34]. Exposure to harmful stimuli can result in excessive ROS production in the brain, which will lead not only to a decrease in the activity of antioxidant enzymes, such as SOD but also to an increase in the contents of endogenous oxidative damage-related products, such as MDA, which is a product of lipid peroxidation [35]. Excessive MDA production in the brain will further reduce SOD levels, resulting in a vicious cycle of oxidative stress in the central nervous system. If excess ROS cannot be removed from the brain, microglia will be activated and release pro-inflammatory factors including IL-1β, IL-6, and TNF-α through downstream signaling pathways, thereby triggering neuroinflammation and eventually causing brain tissue damage and impairing cognitive function [36, 37]. Lead exposure in rats early in life leads to cognitive decline, accompanied by upregulated oxidative stress and inflammatory responses in the brain [38]. Consistent with this observation, we found that SD during late pregnancy resulted in oxidative stress and neuroinflammation in the hippocampus and prefrontal cortex in aged offspring, as evidenced by the increased levels of ROS and MDA, decreased SOD activity, and upregulation of the levels of Iba-1 and IL-1β, IL-6, and TNF-α. EE could improve cognitive impairment by inhibiting neuroinflammation and oxidative stress [39]. For example, an enriched environment was reported to improve middle cerebral artery occlusion-induced cognition impairment in Sprague–Dawley rats by suppressing neuroinflammation and oxidative stress and increasing the expression levels of synaptic plasticity-related proteins in the hippocampus [25]. Similarly, our current results showed that long-term exposure to EE suppressed oxidative stress and neuroinflammation in the hippocampus and prefrontal cortex of aged offspring induced by SD during late pregnancy.

### EE reversed the MSD-induced decline in synaptic protein levels

The synaptic plasticity in the hippocampus and prefrontal cortex plays a critical role in cognitive function [40]. PSD-95 is a post-synaptic protein involved in long-term potentiation as well as synaptic connectivity and maintenance [41]. SYN is a specific calcium-binding glycoprotein widely existed in the pre-synaptic vesicle membrane and plays a role in synaptic plasticity by regulating synaptic structure and function in addition to neurotransmitter release [42]. Basic studies have shown that PSD-95 and SYN knockout mice exhibit impaired synaptic morphology and function accompanied with learning and memory deficits [43, 44]. In clinical studies, the levels of PSD-95 and SYN were significantly decreased in postmortem brain tissue of AD patients compared with those of controls [45]. This clearly indicates that PSD-95 and SYN are critical for synaptic plasticity, synaptic integrity, and cognitive function [46, 47]. In the present study, the expression levels of PSD-95 and SYN were decreased in the hippocampus and prefrontal cortex of offspring mice in the MSD group when compared to those in Control animals, suggesting that exposure to MSD can lead to deficits in synaptic plasticity in aged offspring. Furthermore, EE can ameliorate impaired synaptic plasticity caused by exposure to adverse environments early in life. For example, an enriched environment reportedly increased the levels of synaptic plasticity-associated proteins such as BDNF, PSD-95, and SYN in elderly Sprague–Dawley rats subjected to prenatal mobile phone exposure [48]. Similarly, our results showed that long-term EE reversed the downregulated levels of PSD-95 and SYN in aged offspring CD-1 mice of dams that underwent SD during late pregnancy, suggesting that EE may protect cognitive function by improving synaptic plasticity.

### EE attenuated mitochondrial biogenesis dysfunction by activating the Sirt1/PGC-1α pathway

Impaired mitochondrial biogenesis is associated with neuroinflammation, oxidative stress, and synaptic dysfunction [49]. PGC-1α is thought to be a central regulator of mitochondrial biogenesis [50]. The overexpression of PGC-1α can suppress neuroinflammation by regulating microglial polarization [51]; reduce oxidative stress by regulating the redox balance, such as by scavenging ROS and increasing the activities of antioxidant enzymes such as glutathione (GSH) and SOD [52]; and regulate the expression levels of synapse-associated proteins [53]. Furthermore, Sirt1, a histone deacetylase, can modulate mitochondrial biogenesis by modulating PGC-1α [54]. Chronic cerebral hypoperfusion can induce cognitive dysfunction in Sprague–Dawley rats, accompanied by the downregulation of the Sirt1/PGC-1α pathway, impairment of mitochondrial biogenesis, neuroinflammation, and oxidative stress [18]. Similarly, in the present study, we found that MSD led to a decrease in Sirt1 and PGC-1α levels in the hippocampus and prefrontal cortex of older offspring CD-1 mice. Interestingly, one of the mechanisms by which EE improves neurological function in the brain may be by enhancing or stimulating mitochondrial biogenesis [55]. We have previously demonstrated that EE counteracts alterations in mitochondrial quality control patterns, such as mitochondrial biogenesis, mitophagy, and dynamics, resulting from prenatal inflammatory stimulation in older offspring [23]. Consistent with previous results, we showed here that EE can reverse the decline in the levels of Sirt1 and PGC-1α in the hippocampus and prefrontal cortex of older offspring mice induced by MSD during late pregnancy. These results suggest that EE can ameliorate MSD-mediated mitochondrial biogenesis dysfunction in aged offspring mice by activating the Sirt1/PGC-1α pathway.

Despite the importance of our findings, our study had some limitations. First, we did not examine changes in mitochondrial morphology and other functions such as mitochondrial dynamics and autophagy; secondly, we did not use pharmacological modalities, such as Sirt1 blockers, to inhibit the Sirt1/PGC-1α pathway to observe the effects of EE; thirdly, a control+EE group was did not set for further comparison; thirdly, we did not set up a control group of young mice to observe the effects of sleep deprivation in late pregnancy on aging-related cognitive dysfunction and the putative ameliorative effect of an enriched environment in this process; finally, we did not use immunohistochemistry to quantitatively analyze the effect of EE on the expression levels of synaptic proteins and Iba-1 in different subregions of the hippocampus.

## CONCLUSIONS

**Figure 8:**
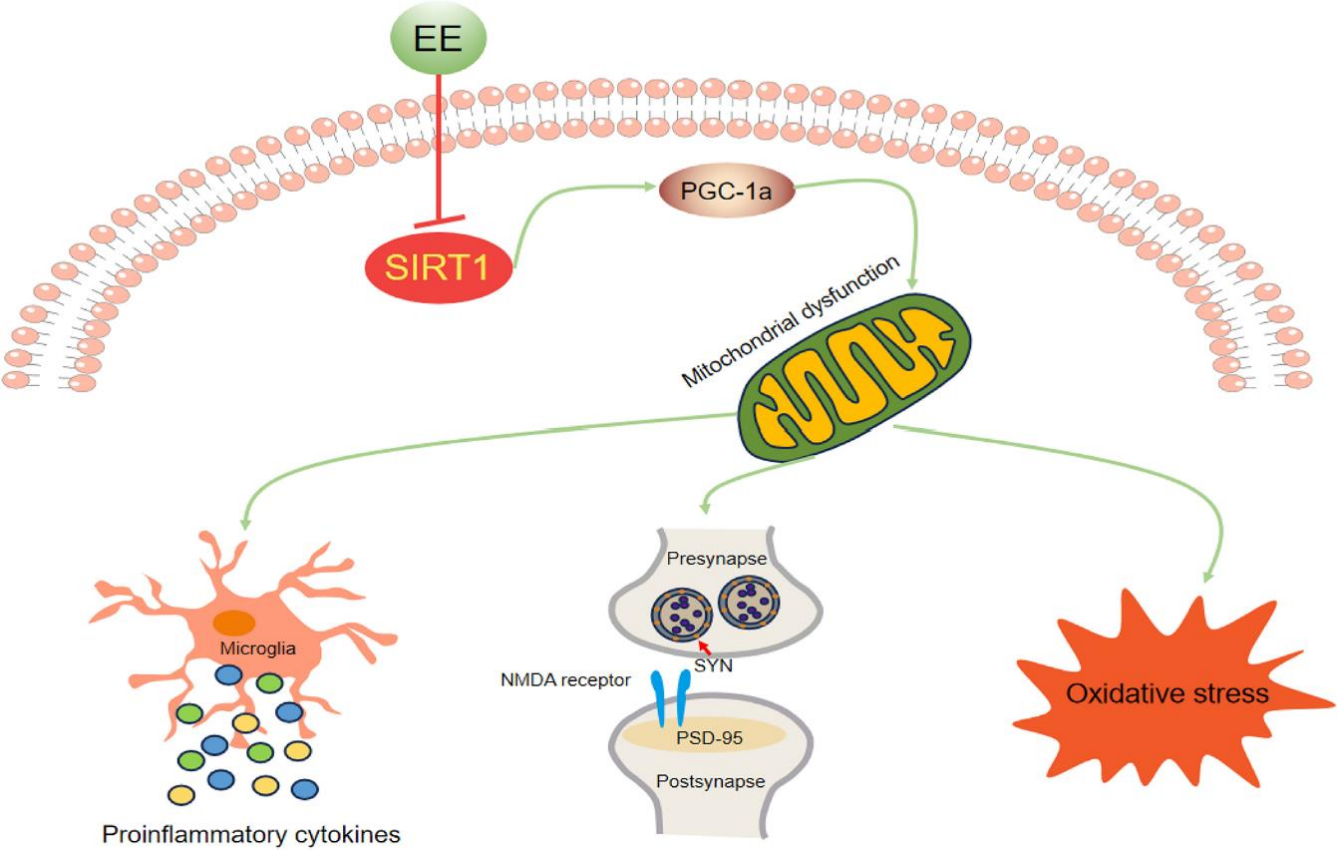
Schematic representation of the mechanisms underlying the inhibitory effect of EE on MSD-induced cognitive impairment in aging CD-1 mice.

We found that long-term EE ameliorated late-pregnancy SD-induced cognitive impairment, attenuated neuroinflammation and oxidative stress, and upregulated synaptic protein levels in aged offspring mice. These effectively beneficial effects were associated with the attenuation of mitochondrial biogenesis dysfunction via the activation of the Sirt1/PGC-1α pathway (Figure 8). These findings suggest that long-term EE may be an effective non-pharmacological intervention for early-life stress-induced cognitive dysfunction, and the Sirt1/PGC-1α signaling pathway may be a promising therapeutic target for this condition.

## MATERIALS AND METHODS

### Animals

**Figure 9:**
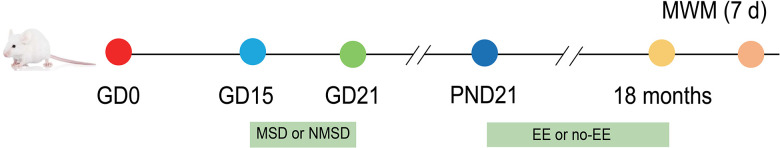
**Experimental procedure and treatment schedule.** GD: gestational day; PND: postnatal day; MWM: Morris water maze test; MSD: maternal sleep deprivation; NMSD: no MSD; EE: environmental enrichment.

2-month-old CD-1 mice were obtained from Beijing Vital River Laboratory Animal Technology Co., Ltd (Beijing, China). After two weeks of environmental adaptation, the mice were mated at a male: female ratio of 1:2. Pregnant female mice were randomized into a control group and a SD group. Female mice in the SD group were subjected to SD during late pregnancy, whereas no treatment was administered to animals in the control group. At 21 days of age, the male and female offspring were divided into the following three groups respectively: control group, MSD group, and MSD+EE group. Mice in the MSD+EE group were given an enriched environment from 21 days to 18 months after birth. During the experiments, all animals had access to water and food ad libitum and were reared at a temperature of 22-25° C and a humidity of 50 ± 5%. In addition, a 12-hour/12-hour light/dark cycle was provided with lights on at 8:00 am (Figure 9).

### Exposure to sleep deprivation

Female CD-1 mice were performed to SD from 12:00 to 18:00 h daily between day 15 and day 21 of pregnancy. SD was administered using a SD apparatus (BW-NSD404, China). The apparatus worked uninterruptedly at a speed of 0.5 m/min to keep mice awake during the deprivation period as previously reported [9].

### Exposure to an enriched environment

Offspring CD-1 mice were subjected to long-term EE from 21 days to 18 months of age. Seven or eight mice were placed in a large cage (52 × 40 × 20 cm^3^) containing colored toys such as plastic tunnels, stairs, running wheels, and wooden houses. The environmental enrichment cages provided more opportunities for social interaction and space for somatosensory and cognitive stimulation as well as physical activity [56]. In contrast, the standard cages (36 × 18 × 14 cm^3^) did not contain any objects, and each housed three mice.

### Morris water maze test

The Morris water maze test (MWM) was used to assess cognitive performance in mice [31]. The equipment mainly consists of a circular pool containing 22° C water (radius: 75 cm, height: 30 cm) and a target platform of cylindrical shape (radius: 5 cm, height: 24 cm). The training phase included four training sessions per day for a total of seven consecutive days. During this period, there is a target platform 1 cm underwater in the target quadrant, and the mice were placed in the water facing the wall and allowed to freely explore for 60 s. When mice found the platform, it was allowed to stay there for 30 s. Each mouse was trained four times a day from different quadrants in order. Mice without finding the target platform within 60 s were led to the platform and also allowed to rest there for 30 s. A video tracking system (ANY-Maze, Stoeling, USA) was applied to record the escape latency (time taken to locate the target platform), the swimming distance, and the swimming velocity of mice. A spatial probe task was conducted 2 h after the last trial of the training phase. For the probe task, removed the target platform, the animals were entered into the water maze from the opposite quadrant of the target quadrant and were given free exploration of 60 s. The percent of time and distance swam in the target quadrant were calculated.

### Assays for ROS, MDA, and SOD contents

Hippocampal and prefrontal cortical tissues were homogenized on ice. After centrifugation (12,000 rpm for 20 min), store supernatant at –80° C. MDA and SOD contents were assayed by the respective kits (Nanjing Jiancheng, China) following the manual. A ROS assay kit (Beyotime Biotechnology, China) with fluorescent probe (DCFH-DA) was employed to determine ROS content. Absorbance was measured in a spectrofluorometer (emission 525 nm, excitation 488 nm).

### Measurement of proinflammatory cytokine levels

The levels of IL-1β, IL-6, and TNF-α in the hippocampus and prefrontal cortex were assessed by the ELISA kits (MyBioscience, Inc., USA) following the operation instructions, respectively.

### Real-time fluorescent quantitative PCR (qPCR)

Total RNA was obtained from hippocampal or prefrontal cortical tissues using TRIzol reagent (Life Technologies, USA). Then the PrimeScript^TM^ RT reagent Kit with gDNA Eraser (Takara Bio Inc., Japan) was utilized to reverse transcribe RNA into cDNA. The qPCR mixture contained 2× SYBR Green Mix, forward and reverse primers, cDNA, and RNase-free water. A Real-Time PCR System (Thermo Fisher Scientific, USA) was employed for fluorescent quantitative PCR reactions. The analytical method used in this experiment was relative quantification study, calculated as 2^-ΔΔCt^. The primers used for qPCR are described below (Table 1).

**Table 1:** The primers used for qPCR.

**Gene**	**Forward primer (5’→3’)**	**Reverse primer (5’→3’)**
β-actin	AGTGTGACGTTGACATCCGT	TGCTAGGAGCCAGAGCAGTA
Psd95	GCTCCCTGGAGAATGTGCTA	TGAGAAGCACTCCGTGAACT
Syn	GCCTACCTTCTCCACCCTTT	GCACTACCAACGTCACAGAC
Sirt1	TAATGTGAGGAGTCAGCACC	GCCTGTTTGGACATTACCAC
Pgc-1α	TGTGACTGGGGACTGTAGTA	AGAGCAGCACACTCTATGTC
Iba-1	ATTCCTCGATGATCCCAAAT	CCAAGTTTCTCCAGCATTCG

### Western blotting

In the hippocampus and prefrontal cortex, western blot was examined for the protein expression levels of PSD-95, SYN, Iba-1, PGC-1α, and Sirt1. Briefly, hippocampal or prefrontal cortical tissues were lysed in RIPA lysis buffer (Beyotime Biotechnology, China) and then collect the supernatant after centrifugation. Protein samples were treated with SDS–PAGE and transferred to PVDF membranes (Millipore, USA), then blocked in 5% skimmed milk for 2 h at room temperature. The incubation was first carried out with the primary antibody against Iba-1 (1:200, Santa Cruz, USA), SYN (1:2,000, Bioss, China), PSD-95 (1:1,000, Abcam, UK), PGC-1α (1:1,000, Abcam, UK), and Sirt1 (1:500, Santa Cruz, USA) overnight at 4° C. Then incubated with the horseradish peroxidase-conjugated secondary antibodies (anti-rabbit/mouse IgG; 1:20,000; Zsbio, China) for 1.2 h, respectively. Protein band intensities were quantified using ImageJ (Media Cybernetics, USA).

### Statistical analysis

Data were presented as means ± standard error of the mean (SEM) and conformed to the chi-square test for normality. Differences among groups were assessed by repeated-measures analysis of variance (ANOVA) or two-way ANOVA with Tukey’s *post hoc* test. All data were analyzed in GraphPad Prism 8.0 and the statistical significance level was set at *P*-value < 0.05.

## Supplementary Material

Supplementary Figures
