# Supplementary material for: An enriched environment ameliorates maternal sleep deprivation-induced cognitive impairment in aged mice by improving mitochondrial function via the Sirt1/PGC-1α pathway
Source: Aging (Albany NY). 2024 Jan 16;16(2):1128–44. doi: 10.18632/aging.205385 (PMC10866428; doi:10.18632/aging.205385)
Supplement: Supplementary Figures [file aging-16-205385-s001.pdf]

SUPPLEMENTARY FIGURES

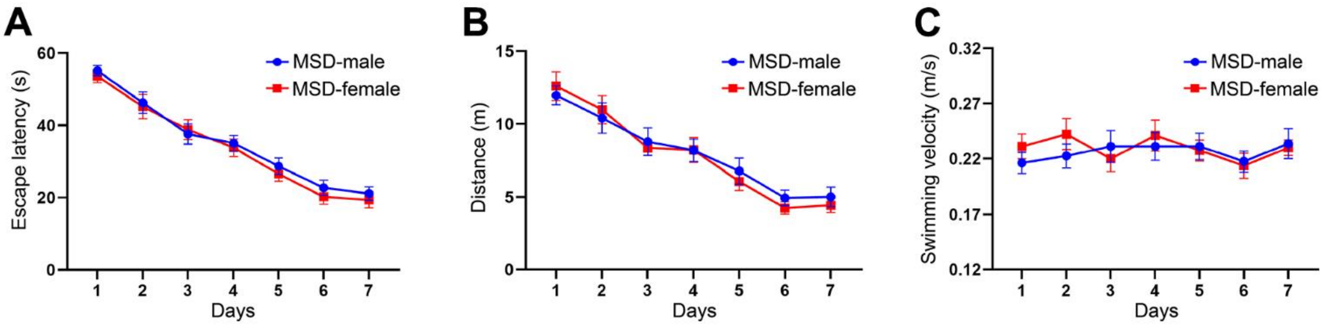

Supplementary Figure 1. The escape latency, distance, and swimming velocity of the learning period of Morris water maze (MWM) test in the MSD groups. The escape latency (**A**), distance (**B**), and swimming velocity (**C**) between male and female mice were not statistically different in the MSD groups. There are 8 male and 8 female mice in the MSD group. Error bars = SEM. MSD, maternal sleep deprivation.

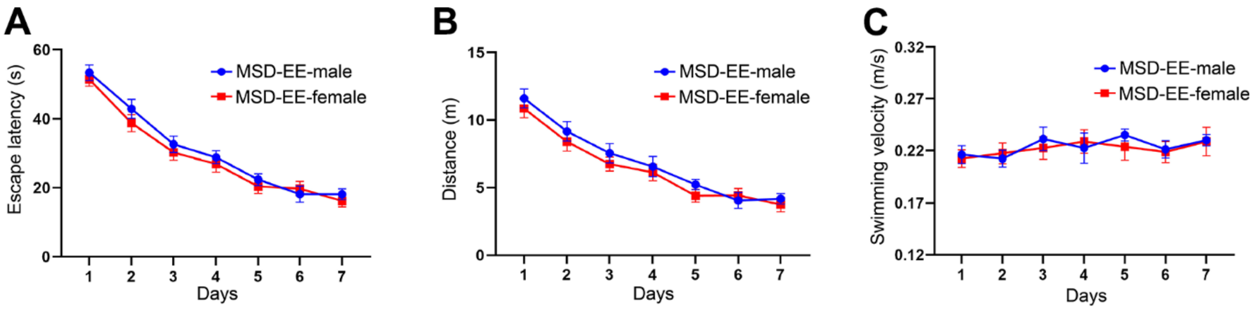

Supplementary Figure 2. The escape latency, distance, and swimming velocity of the learning period of Morris water maze (MWM) test in the MSD-EE groups. The escape latency (**A**), distance (**B**), and swimming velocity (**C**) between male and female mice were not statistically different in the MSD-EE groups. There are 8 male and 8 female mice in the MSD-EE group. Error bars = SEM. MSD, maternal sleep deprivation; EE, enriched environment.
